# Unraveling the “Greener Pastures” Concept: The Phenomenology of Internationally Educated Occupational Therapists

**DOI:** 10.1177/15394492231205885

**Published:** 2023-10-12

**Authors:** Roi Charles Pineda, Brenda Abad-Pinlac, Daryl Patrick G. Yao, Fides Nadine Raya B. Toribio, Staffan Josephsson, Michael Palapal Sy

**Affiliations:** 1 Katholieke Universiteit Leuven, Belgium; 2 Amsterdam University of Applied Sciences, The Netherlands; 3 Tokyo Metropolitan University, Japan; 4 University of Illinois at Chicago, USA; 5 Handicap International—Humanity & Inclusion, Makati, Philippines; 6 Karolinska Institutet, Huddinge, Sweden; 7 Zurich University of Applied Sciences, Winterthur, Switzerland; 8 University of the Philippines Manila, Philippines

**Keywords:** cultural/culture sensitivity, international education, phenomenology, international migration

## Abstract

The Philippines is one of the major labor exporters for health care workers in the world and occupational therapists are the second most likely professionals behind nurses to work in “greener pastures” overseas. This phenomenological study describes the migration experiences of Philippine-trained occupational therapists working in high-income, Anglophone countries. Researchers conducted online interview for 15 participants who were previously/currently unemployed/underemployed. Following an inductive approach to qualitative data analysis, themes were drawn from the migrant therapists’ lived experiences. Four themes describe the migration experiences of Filipino occupational therapists: (a) realities of being an occupational therapist in the Philippines, (b) contradictions faced by Filipino occupational therapists upon migration, (c) when the pastures overseas are not greener, and (d) when the pastures overseas are indeed greener. The study contributes to the labor migration discourse in occupational therapy and the critical examination of the idea of “greener pastures.”

## Plain Language Summary

The Migration and Deskilling of Filipino Occupational Therapists

This study provides stories of Filipino occupational therapists who were underemployed as they migrated to developed and English-speaking countries. We interviewed 15 people online to explore their experiences of migration, being underemployed, and working in a foreign country with a different culture of working. Results of the study revealed four ideas: (a) reality of being an occupational therapist in the Philippines, (b) contradictions faced by Filipino occupational therapists upon migration, (c) barriers faced by migrant occupational therapists, and (d) successes experienced by migrant occupational therapists. This study contributes to the ongoing research on occupational therapy, labor migration, and overseas Filipino workers.

## Introduction

Due to the continued population growth and longer life expectancy worldwide, the World Health Organization (WHO, 2016) estimates an 18-million health care workforce deficit by 2030. This health care worker shortage crisis resulted in an increased reliance on outsourced workers from other countries as professionals from low- and middle-income countries strengthened the health care workforce in high-income nations over the past decade (Organisation for Economic Co-operation and Development [OECD], 2019; WHO, 2014). The Philippines is a major source country for labor exports, ranking first in exporting nurses and tenth in exporting physicians to OECD member countries (Cheng, 2009; OECD, 2020). Among health care professionals in the Philippines, occupational therapy practitioners are the second most likely group, following nurses, to migrate out of the country (Lorenzo et al., 2011). Yet research investigating the migration experiences of migrant Filipino occupational therapists is lacking.

The reasons underlying the decision to migrate are complex. Push factors are unfavorable circumstances in the place of origin (e.g., job insecurity) that reinforces emigration, whereas pull factors are things that attract the immigrant to move to the destination country (e.g., higher salary; Lee, 1966; Lorenzo et al., 2007; Toyin-Thomas et al., 2023). In addition, other variables such as source/destination country’s immigration policies can shape migration decisions (Castro-Palaganas et al., 2017). Nonetheless, work overseas is an appealing option for Filipinos who desire better living standards and this is facilitated by the Department of Migrant Workers (formerly Philippine Overseas Employment Administration). Initially, a temporary solution for the economic challenges in the 1970s (Christ, 2016), institutionalized labor exportation has become a significant contributor to the Philippine economy. Approximately 10.2 million Filipinos or 10% of the population reside and work overseas (Commission on Filipinos Overseas, 2013). In 2021, their remittances reached US$31.4 billion, accounting for 8% of the country’s gross domestic product (Bangko Sentral ng Pilipinas, 2021).

The economic incentive of labor exportation prompted the glamorization of Overseas Filipino Workers (OFWs) as *bagong bayani* (modern-day heroes) who are celebrated for their contribution to nation-building through remittances (Brush & Sochalski, 2007). Countless success stories of OFWs have deeply ingrained the aspiration to seek the greener pasture abroad in Philippine society. The “greener pasture” concept represents opportunities to acquire the means for upward social mobility (e.g., money and work experience) that is perceived to be lacking in the Philippines but abundant abroad (Ronquillo et al., 2011). Filipino youth are encouraged to pursue careers in high-demand fields and are particularly drawn to health care professions, which offer better opportunities for well-paying jobs abroad (Castro-Palaganas et al., 2017; Christ, 2016). However, the negative facets of migration are often overlooked. Unceasing emigration leads to brain drain (i.e., depletion of a nation’s skilled workforce necessary for development) and exacerbation of existing health care workforce shortage (WHO, 2014). In addition, skilled immigrants may face racial discrimination in the forms of verbal abuse, harassment, work segregation, and restricted professional mobility in Australia (Kosny et al., 2017), Canada (Baptiste, 2015), the United Kingdom (Kesler & Safi, 2017), and the United States (Al Achkar et al., 2023). Because of the challenges they face in these countries, some immigrants are compelled to accept lower level positions than their qualifications, resulting in occupational downward mobility known as “deskilling” (Gotehus, 2021).

The top four destination countries for Filipino health professionals are the United States, Canada, Australia, and the United Kingdom (Castro-Palaganas et al., 2017). The first hurdle that internationally educated occupational therapists (IEOTs) face in these countries is a registration eligibility assessment, which is based on academic credentials, fieldwork hours, and English-language proficiency (see Table 1). Those who fall short on these assessments may have to take courses to offset deficiencies in their credentials. Beyond these, IEOTS in Australia are required to undergo a supervised practice period before receiving registration with the Occupational Therapy Board (Australian Health Practitioner Regulation Agency, 2023). Studies on the experiences of IEOTs in the United States, the United Kingdom, and Canada revealed that many struggle with qualification mismatch between source and destination countries (Amerih & Hersch, 2009; Mulholland et al., 2013). Although countries such as Canada have programs to support the transition of IEOTs to practice in their destination countries (Baptiste et al., 2020), the rigorous process of assessment and practice registration may lead to some IEOTs succumbing to unemployment/underemployment and eventual deskilling.

**Table 1.** Qualifications for Internationally Educated Occupational Therapists.

Qualifications	Australia	Canada	United Kingdom	United States
Minimum academic credentials	Bachelor’s degree in occupational therapy^a^	Master’s degree in occupational therapy	Bachelor’s degree in occupational therapy^a^	Master’s degree in occupational therapy
Minimum fieldwork hours	1,000	1,000	1,000	960
Proof of English proficiency	TOEFL and IELTS, among others	TOEFL and IELTS, among others	TOEFL or IELTS	TOEFL or Cambridge Assessment English
IEOT eligibility determination and cost	Desktop assessment by the Occupational Therapy Council of Australia (1,500 AUD)	Substantial Equivalency Assessment System by the Association of Canadian Occupational Therapy Regulatory Organizations (4,000 CAD)	Registration eligibility through the international route by the Health and Care Professions Council (539.65 GBP)	Occupational Therapy Eligibility Determination by National Board of Certification in Occupational Therapy (NBCOT; US$850)
Examination and cost	None	National Occupational Therapy Certification Examination (755 CAD)	None	NBCOT certification examination (US$515)
Registration	Occupational Therapy Board of Australia	Provincial regulator; requirements vary for each province	Health and Care Professions Council	State regulator; requirements vary for each state
Link	https://www.otcouncil.com.au/assessment/assessment-in-australia/	https://acotro-acore.org/registering-in-canada/were-you-educated-outside-of-canada/	https://www.hcpc-uk.org/registration/getting-on-the-register/international-applications/	https://www.nbcot.org/get-certified/eligibility

*Note*. Philippine-trained occupational therapists have a minimum academic credential of bachelor’s degree in occupational therapy, with at least 1,200 fieldwork hours. IEOT = internationally educated occupational therapist; TOEFL = Test of English as a Foreign Language; IELTS = International English Language Testing System.

a Degree program must be recognized by the World Federation of Occupational Therapists.

Given the limited research on IEOTs, exploring personal accounts of immigrant occupational therapists who are/were unable to secure jobs befitting their qualification (i.e., unemployed/underemployed) in their destination country may provide a balanced perspective on the glamorized image of migrant skilled workers. This research can also inform policies and programs to encourage the retention and migration of Filipino occupational therapists. Therefore, our study aimed to describe the lived experiences of Philippine-educated occupational therapists living and working abroad. Specifically, we sought to elucidate (a) their motivations for labor migration, (b) the advantages of living/working abroad, and (c) the challenges of leaving the Philippines and becoming immigrants.

## Method

This study used a qualitative phenomenological research design to describe the lived experiences of individuals about a phenomenon from the participants’ own descriptions (Creswell, 2013). Specifically, the phenomenological approach used was drawn from Moustakas (1994) where the focus remained on the descriptions of the participants’ experiences, setting aside the researchers’ experiences and views. More than describing the process of migration of selected individuals who experienced unemployment/underemployment, meanings and their interpretations were generated from their lived experiences as migrant Filipino occupational therapists, producing *textural descriptions* (what participants experienced) and *structural descriptions* (how they experienced it within contexts and situations) drawn from in-depth interviews. The study’s protocol was approved by the institutional ethics board of the University of the Philippines Manila.

### Participants

A purposive sample of participants was recruited based on the following inclusion criteria: (a) graduated from an occupational therapy degree program in the Philippines; (b) migrated to the United States, Canada, Australia, or the United Kingdom at least 1 year prior to the interview; and (c) are/were unemployed or underemployed in their destination country (have/had a job that did not match qualifications acquired in the Philippines). Filipinos expect labor migrants to have good, high-paying jobs. Those who do not attain such jobs may experience negative emotions (e.g., shame and low self-worth) and may be secretive of this perceived failure (Kelly & Lusis, 2006). To reach the intended participants better, we used chain-referral sampling, starting from individuals known to the researchers. All participants digitally signed a written informed consent prior to enrollment into the study.

### Instrument and Procedure

Guide questions and possible probes for the semi-structured interview (Table 2) were developed and refined from a pilot study (Pineda & Sy, 2021). Two researchers, R.C.P. and B.A.-P., conducted individual online video interviews that lasted 60 to 90 min each. Interviews were done in a mix of English and Filipino (as preferred by the participants) and were audio-recorded for transcription. The interview approach was conversational, using the guide questions to initiate the conversation and allowing the participants’ own responses to direct the conversation, especially for potentially sensitive topics (e.g., unemployment experience). Probes helped clarify and elaborate participants’ responses and explore topics that were not spontaneously disclosed.

**Table 2.** Interview Guide Questions.

Guide question	Possible probes
1. Tell us about your life and work in the Philippines prior to migrating.	• Brief work history • Circumstances and reasons for considering migration
2. How did you migrate to [destination country]?	• Involvement of recruitment agency • Reason for choosing to migrate to [destination country] • Timeline
3. Tell us about your life and work situation in [destination country] from when you arrived to the present.	• Brief work history in [destination country] • Process of licensing/registration for occupational therapists in [destination country] (e.g., supports for and barriers to obtaining license/registration, timeline) • Transition to and integration into [destination country] • Prospect of upward occupational mobility • Negative experiences (e.g., discrimination)
4. Tell us about the period when you were unemployed or underemployed in [destination country].	• Circumstances and reasons for unemployment or underemployment in [destination country] • Impact (e.g., emotional status, deterioration of occupational therapy knowledge/skills) • Timeline
5. Compare your life and work in the Philippines with your current life and work in [destination country].	• Keeping connection with families and friends back in the Philippines • Advantages and disadvantages of being an immigrant worker in [destination country] compared with being in your home country

### Data Analysis

Analysis and interpretation of textual data followed an inductive approach (Creswell, 2013). Each interview transcript was assigned to two researchers who independently read and reread the interview transcripts, took notes, and formed initial codes. Coding was facilitated using ATLAS.ti7. Thereafter, the team came together to consolidate these codes and organized 302 unique codes into themes (three themes initially). Through an iterative process, the researchers collectively reorganized themes, developed subthemes, constructed a qualitative narrative, and interpreted the findings. Any disagreements were discussed as a team until a consensus was reached.

### Rigor and Trustworthiness

To ensure research quality, strategies outlines by Elo et al. (2014) were employed. *Credibility* was demonstrated through semi-structured interviews following a phenomenological approach. To minimize biases, positionality was declared by the researchers. All are occupational therapists who have qualitative research experience. S.J. has published several migration studies. R.C.P., B.A.-P., D.P.Y., F.N.T., and M.P.S. are Filipinos and, except for F.N.T., are migrants themselves. Moreover, we employed critical reflexivity by being aware of our subjective thoughts and by pairing researchers who personally knew the participants with another researcher unfamiliar to the participants during data analysis. *Dependability* was maintained by holding regular meetings (accompanied by written minutes for audit trailing) to assess adherence to methodological procedures and ethical guidelines. *Confirmability* was established through regular consultations among researchers. In addition, member checking was done by holding follow-up interviews with seven participants who consented to a second interview and for documenting their feedback. *Transferability* was addressed by providing a detailed outline of the study’s steps and describing salient findings to aid readers in assessing its applicability in other contexts.

## Results

Fifteen participants were interviewed (Table 3). Participants’ age ranged from 23 to 47 years, with 67% identifying as female. Most practiced occupational therapy in the Philippines before emigrating (73%). The sample included both recent (<5 years) and long-term (>10 years) immigrants, including those who have obtained citizenship in their destination country. At the time of the interview, only five participants were working as occupational therapists.

**Table 3.** Characteristics of Study Participants (*N* = 15).

Pseudonym	Age range, years	Sex	Work in the Philippines prior to migration	Current country of residence	No. of years living overseas	Current employment
Celine	20–29	Female	Occupational therapist	Australia	<5	Occupational therapist
Lorna	40–49	Female	Occupational therapist	Australia	>20	Occupational therapist
Angeline	30–39	Female	Occupational therapist	Canada	5–10	Non–health care
Nicole	30–39	Female	None	Canada	5–10	Non–health care
Joyce	30–39	Female	None	Canada	5–10	Occupational therapist
Kenneth	30–39	Male	None	Canada	< 5	Therapy assistant
Ester	30–39	Female	Occupational therapist	United Kingdom	5–10	Therapy assistant
Sharon	40–49	Female	Occupational therapist	United Kingdom	11–20	Health care facility manager
Vangie	40–49	Female	Occupational therapist	United Kingdom	11–20	Caregiver
Dante	40–49	Male	Occupational therapist	United Kingdom	5–10	Therapy assistant
Wilfredo	40–49	Male	Occupational therapist	United Kingdom	11–20	Therapy assistant
Trisha	20–29	Female	Occupational therapist	United States	<5	Non–health care
Zara	20–29	Female	None	United States	<5	Caregiver
Ronnie	30–39	Male	Occupational therapist	United States	5–10	Occupational therapist
Manny	40–49	Male	Occupational therapist	United States	11–20	Occupational therapist

Four themes described the migration experiences of Filipino occupational therapists: realities of being an occupational therapist in the Philippines, contradictions faced by Filipino occupational therapists upon migration, when the pastures overseas are not greener, and when the pastures overseas are indeed greener. These themes, along with several subthemes, are summarized in Table 4.

**Table 4.** Themes and Subthemes Describing the Lived Migration Experiences of Filipino Occupational Therapists.

Themes	Subthemes
1. Realities of being an occupational therapist in the Philippines	• Socioeconomic realities
	• Professional realities
2. Contradictions faced by Filipino occupational therapists upon migration	• Training
	• Professional roles
	• Employment situation
	• Meaning of success
3. When the pastures overseas are not greener	• Agential dynamics in the new workplace
	• Unsettling detour to employment
	• Ugly truths of being an immigrant
4. When the pastures overseas are indeed greener	• Resilience amid migration
	• Fruits of migration

### Realities of Being an Occupational Therapist in the Philippines

This theme highlights the traditional perception of the “greener pasture” idea that drove participants to emigrate and is categorized into two subthemes. First are the socioeconomic realities of life in the Philippines. Strong familial ties and financial insecurity from inadequate salaries played significant roles in their decision to migrate. They harbored a sense of duty to either support their families in the Philippines or reunite with their families already living overseas. As a single parent, Sharon stressed, “Had I stayed in the Philippines, I would not have been able to support my parents and siblings while providing for . . . my kids.” Similarly, by working overseas, Angeline fulfilled her familial obligations while “still having enough to build savings and maintain a comfortable lifestyle.” All participants acknowledged the pull of significantly higher salaries overseas, regardless of the work. Illustrating this point well, Ronnie disclosed that his annual income in the Philippines is comparable to 3 weeks’ income as an occupational therapist in the United States. Meanwhile, Wilfredo could earn his annual income in the Philippines within 4 months of working as an occupational therapy assistant in the United Kingdom. The participants also pointed out superior health care benefits overseas, to which Manny lamented, “you’ll get buried in debt if you or your family gets sick in the Philippines.”

Emigrating out of the Philippines was sometimes a family decision. Lorna revealed, “I was getting pressured by my mom [to work overseas] although I didn’t want to. But that is the culture, isn’t it? We’re expected to work abroad to earn more.” For participants of migrant parents, they migrated to reunite or move with their families abroad. According to Nicole, “My mom is here [in Canada] and she wanted me to come. My mom and I have been apart since I was five. So, she wanted us to be reunited.” Considering where they would have been, supposing their parents were not overseas, the participants had differences in opinion. Nicole shared, “Because of better opportunities overseas, even if my mom were not [in the US], I think the natural course would still be to leave the Philippines . . .” In contrast, Zara declared, “I probably would not have [migrated to the USA] if my family were in the Philippines . . . because I cannot imagine living away [from them].”

Participants were also confronted by professional realities in the Philippines, which are characterized by a labor export–oriented occupational therapy education, limited opportunities for professional growth, and heavy workload. Ronnie recalled his experience as a student,

We were conditioned [in Philippine universities] to go to the USA . . . and earn the almighty dollar . . . . Many universities use the opportunity to go abroad as a marketing strategy to attract students into health science courses.

The conditioned predisposition to migrate is further strengthened by the bleak landscape of occupational therapy practice in the Philippines. They observed that private pediatric practice was emphasized in the Philippines. However, even when working in pediatric practice, Trisha commented, “I cannot see how I would grow [as a clinician]. To begin with, I could not find any mentorship. How can I improve my clinical skills?” While Ronnie disagreed about the lack of availability of mentorship, he remarked about the limited availability of certification opportunities: “Unlike in the USA, there are no programs in the Philippines that will make you, for example, a certified hand therapist.” Angeline concurred with this comment, “Many desirable certifications cost a lot of money and the courses are only offered abroad.” Finally, therapists in the Philippines are often inundated by work. “Oftentimes, there is too much work that I need to extend my working hours just to finish everything . . . and that is without extra pay,” Ester recalled.

### Contradictions Faced by Filipino Occupational Therapists Upon Migration

This theme explores the contrasting experiences of participants as they navigate the process of *becoming* and *being* an occupational therapist seeking greener pastures. They encountered contradicting experiences in training, professional roles, employment situations, and the meaning of “success.” Some participants had positive experiences, perceiving Filipino therapists as globally competitive and well trained. Kenneth remarked, “Our occupational therapy education [in the Philippines] is at par with international standards. My colleagues are sometimes surprised that I can perform, what they consider ‘advance’ skills.” Zara added, “We are trained . . . and it is in our culture to show genuine care and empathy, which Americans appreciate.” Other participants were told that their education and experience were insufficient. The experiences of therapists ranged from minor inadequacy in training, such as “not [being] trained to deal with culturally diverse clients,” (Lorna) to invalidation of education and work experience from the Philippines. Vangie divulged that she had to take courses because her education was “inadequate for UK standards.” Similarly, Angeline mentioned, “Only my work experience in the USA was considered in Canada while my more extensive experience in the Philippines was disregarded.”

The multifaceted professional roles undertaken by IEOTs as migrant health care workers also presented contradictions. They acknowledged the significance of professional boundaries and collaboration but also were confronted with the limitations and compartmentalization of occupational therapy roles and expectations to engage in nonclinical tasks. Kenneth described, “Professional roles are highly delineated [in Canada]. Occupational therapists are restricted to motor skills training . . . I was reminded not to encroach on other professions’ domain.”

Another contradiction exists regarding the employability of IEOTs. Securing employment outside occupational therapy was often considered easier compared with occupational therapy–specific positions, which can be more time-consuming to obtain. Furthermore, initial occupational therapy job offers may cater to financial needs but not personal preferences. This employment situation challenges the notion of the “American Dream” due to the reality of fluctuating job demands and the possibilities of human trafficking. As Ronnie disclosed,

The agency that first brought us to the USA was bordering human trafficking because when we were still in the Philippines, the agency promised us [many good things] . . . But once you arrive [in the USA] they could do almost anything to you because . . . we didn’t have a choice . . . . If we didn’t accept the agency’s offer, we’ll be forced to return to the Philippines.

Ronnie’s experience also unearthed the discussion on the meaning of “success.” While success abroad is associated with heightened job satisfaction and formation of professional identity, the meaning of success can also mean the following: doing the bare minimum to get the job done and seeing success in the gratitude of clients. Continuing Ronnie’s sharing, he compared,

It’s insurance driven [occupational therapy practice in the USA] . . . quantity over quality. They do not even use standardized test for quality assurance. They use it to get paid . . . I would still say that my practice in the Philippines was more fulfilling. Filipino patients are grateful when receiving therapy . . . you know they are because they know [therapy] is a luxury.

### When the Pastures Overseas Are Not Greener

This theme counters the “greener pasture” notion and highlights the obstacles faced by Filipino occupational therapists upon migrating. Subthemes within this theme encompass the agential dynamics in the new workplace, the unsettling detour to employment, and the ugly truth of being an immigrant.

Personal and professional agencies (i.e., control over one’s actions and their consequences) are challenged when adapting to a new workplace. Participants confronted their diminished professional autonomy, resulting from limited job opportunities, while facing clients in destination countries who were more autonomous and proactive. These adjustments were coupled with the need to “fit” to new cultural contexts. Angeline shared her experience of losing control over work location because options were limited to what the employment agency offered. Nicole disclosed how her clients were knowledgeable about informed consent, autonomy, and client-centered care, whereas “in the Philippines, you ‘prescribe’ activities and people just accept it.”

Added to the challenge of relocating to a new country, being a Filipino immigrant with incongruent qualifications in a new professional jurisdiction can be unsettling. While working toward licensing/registration, participants may engage in alternative, sometimes clandestine, ways to generate income. These alternative work arrangements entailed the nonuse of their hard-earned credentials, leading to underemployment, deskilling, and feelings of shame. Some even opted to pursue a different career path and to not return to occupational therapy. Sharon stressed, “not being able to practice my profession for years left me feeling demoralized. I have already missed a lot. I’m competing with younger graduates, underemployed or unemployed people for the same opportunities.” Zara and Nicole resorted to working as a care aide and food service crew, respectively, for survival. Zara felt ashamed when questioned by friends about her job in the United States.

Migrants often disclose the “good” and “positive” experiences while concealing the harsh realities of injustices and discrimination (e.g., racism, ageism). Participants experienced discrimination from both foreigners and fellow Filipinos (crab mentality) as they manage the unequal opportunities, housing issues, workplace bullying, and fiscal challenges due to their immigrant status or foreign credentials. Ester mentioned,

Some British colleagues always comment about how they asked for a pay increase. But instead of increasing their wages, the company recruited overseas workers . . . with lower pay. I felt like I am being blamed for something that is beyond my control.

Vangie shared this feeling of being discriminated against and claimed, “Immigrants like me are still not accorded a fair share of respect, recognition, or consideration.” More explicit racism was experienced by Manny and Ronnie. Manny was told by a patient, “I don’t want to work with you because I won’t understand your English.” Ronnie narrated that all he wanted was to be nice as newly hired personnel, but “an elderly lady gave me the [middle] finger on my first day of work.”

### When the Pastures Overseas Are Indeed Greener

In contrast to the third theme, Theme 4 explores the strategies Filipino immigrants employed to ease their transition, pursue their goals in the new country, and validate the concept of “greener pastures.” Participants coped with the stress of migration by finding a community, maximizing the use of technology, maximizing their language skills, and embodying their morality-based work ethics. Vangie shared that, despite all the setbacks, she decided to “always look for the silver lining.” Manny was proud to say that Filipinos are bilingual and this ability “enables us to communicate abroad . . . you will never be left behind [when working abroad].”

With persistence and the right mindset, the “pastures” overseas can indeed be “greener” for immigrants. Participants experienced numerous opportunities for professional growth, personal security, and privileges. Financial gains were notable, with higher earnings in a shorter period compared with the Philippines. In addition, Ronnie shared that, aside from the income, he can achieve “more freedom to have a [high] quality of life . . . owning your own house and car.” Sharon and Celine revealed that more than the financial part, working overseas afforded them free health care and residence in a safe neighborhood and clean environment, surpassing what they would have in the Philippines.

## Discussion

This phenomenological study uncovered detailed experiences of migrant Filipino occupational therapists and revealed that the diaspora is driven by not only financial security and family reunification but also professional growth and societal pressure for better living standards. Our findings also challenge the “greener pastures” concept, as the paradoxical realities of Filipino migrants are experienced across life dimensions, redefining the meaning of “success” and achievement ideology (e.g., “American Dream”). While there are many advantages to migration, this study reveals the nuanced reality of becoming an occupational therapist abroad.

It was apparent in the participants’ experiences that the life-altering decision to migrate is very personal, informed by a combination of various factors and goes beyond the notion of merely obtaining higher salaries abroad. The significant pay disparity serves as the most obvious pull factor for the participants as the average monthly income of occupational therapists in the Philippines is around US$700 (Carandang & Delos Reyes, 2018), which is about 10% or 18% of what they can earn in the United States (Bureau of Labor Statistics, 2022) or the United Kingdom (National Health Service [NHS] Staff Council, 2023), respectively. Limited work opportunities and unsustainable workload made it even less appealing for participants to stay in the Philippines. In fact, Delos Reyes (2018) reported high rates of burnout among Filipino occupational therapists. Furthermore, the Philippines’ culture of migration (Christ, 2016) put pressure on the participants to migrate, many of whom were conditioned as students to do so.

The pull factor of migration is further reinforced by *familism*, a Filipino value emphasizing the significance of family relationships and their influence on identity, behavior, and life priorities (Morillo et al., 2013). Within this concept, family resources are typically viewed as shared, requiring every member to contribute for the family’s survival. Our results (Theme 1) showed that many participants worked overseas to support their families back home. However, breadwinning can become contradictory to shared family responsibilities and resources. This is the case when only one family member assumes all familial obligations—sometimes by migrating overseas—to financially support themselves as well as their newly established family and/or the family they left behind.

The Philippine government continues the institutionalization of exporting Filipino human resources for health to high-income countries, advertising these qualified health workers overtly as modern-day heroes, but covertly as cheap highly skilled laborers (Cheng, 2009). Our study participants may have migrated for varied reasons and through different pathways. Despite their academic credentials and work experiences from the Philippines, many entered the destination country as temporary workers with lower remuneration, compared with their counterparts with citizenship. Supported by our findings (Themes 2 and 3), the greener pasture concept is also being reinforced by policies set by destination countries where qualifying to become a qualified occupational therapist requires one to encounter systemic structures masked as “competence equivalency.” Promising improved living conditions and opportunities, migration has become a fundamental aspect of Filipino identity that is instilled at a young age. However, our study deglamorizes international migration by highlighting the conflicting realities of life and work situations faced by migrants. The sociological study of Francisco (2009) reveals the flaws in adopting migration strategies as a part of national development as they compromise safety, dignity, and rights. This is evidenced by the work-related abuse incidents against OFWs and the “perpetual” temporary status of immigrant workers confounded by fraudulent immigration consultants (Alcaraz et al., 2021). On the contrary, migrant occupational therapists themselves could also perpetuate the “greener pastures” myth by only often showcasing the “positive” experiences and “sanctioned” expectations to somehow hide the realities of migration.

Emigration trends indicate Filipinos exercising agency toward the ability to migrate freely to another country (Francisco, 2009). However, for Gotehus (2021), this agency does not lead to long-term autonomy, as she demonstrated in a study on Filipino nurses in Norway. Deskilled and underemployed, the participants’ dream of becoming a nurse overseas activates their collective agency underpinned by silent compliance to regulations, bearing exploitative practices, and settling for less. From an occupational science perspective, the occupation of migration entails the feelings of *paghihirap* (suffering) representing the physical and emotional pain caused by extreme *pagsisikap* (hard work; Sy et al., 2023). The study participants understood that they would face difficulties regardless of whether they migrate or not. However, the benefits they could reap from working hard abroad outweigh the potential for suffering, unlike if they stayed in the Philippines. Their decision to move to “greener pastures” is freely chosen and associated risks are endured to fulfill familial duties and achieve belongingness. Based on our findings, the greener pasture concept also galvanizes the notion of “occupational possibilities” (Laliberte Rudman, 2010). In relation to this concept, the occupation of “seeking greener pastures” does not have the same motivations and outcomes for all, but are rather situated and contextualized based on the interplay of existing structures, agencies, and privileges and lack thereof.

Regardless of the World Federation of Occupational Therapists’s (WFOT, 2020) encouraging statements regarding the unique, enriching, and fulfilling experiences of working as an occupational therapist abroad, our study findings revealed that the surge of immigration in search of “greener pastures” among Filipino occupational therapists will likely continue. The contemporary conceptualization of “greener pasture” extends beyond the traditional notion of higher salaries for better life opportunities (Dimaya et al., 2012). This concept encompasses notions of prestige for Filipino health workers abroad, a competitive job market in the health service sector, the reinforcement of familism, and viewing the health science degree as a gateway to the world (Amrith, 2013; Ronquillo et al., 2011). Our findings demonstrate that the “greener pasture” is not merely a destination but an ongoing reality that shapes the Filipino identity, constituted by paradoxical circumstances, overcoming unsettling truths, and reaping the fruits of pain, hard work, and perseverance.

## Limitations and Future Research

We acknowledge that this study had limitations. First, analyzing experiences of 15 participants from four countries cannot generalize the experiences of all immigrants and Filipino occupational therapists. However, it can illuminate realities and nuances of migratory experiences among health care professionals. Second, because none of the researchers have experienced being deskilled or unemployed, there may be aspects of the participants’ experiences that were overlooked, missed, or simplified in spite of the rigorous and iterative analyses employed. Given these limitations, we propose that future research on the topic could explore more specific comparative dimensions of migratory patterns and experiences of occupational therapists in different countries, and investigate the interplay of colonial dynamics within the occupational therapy profession in the Philippines.

## Conclusion

This study sheds light on the lived experiences of migrant Filipino occupational therapists who were underemployed and deskilled as a consequence of migration. Through the four themes generated, our findings revealed a nuanced understanding of migration. Migration is an ongoing reality for many Filipino occupational therapists, motivated by personal and professional aspirations. Given the advantages and disadvantages of migrating, Filipino occupational therapists are constantly caught in the middle of contradicting situations in the migration process, thereby challenging the notion of the “greener pastures.” Our study findings are intended to signpost occupational therapists to reflect on the process of migration by deglamorizing the concept of international migration, unmasking the social realities of migrant health workers, and unraveling the complexities of migration as an occupation. While our findings are limited and cannot be generalized to all migrant Filipino occupational therapists, we hope to contribute to the ongoing discourse on Filipinos working overseas by maintaining a critical stance toward the concept of “greener pastures.”
